# Raman Spectroscopy and Aptamers for a Label-Free Approach: Diagnostic and Application Tools

**DOI:** 10.1155/2019/2815789

**Published:** 2019-04-28

**Authors:** Elisa Scatena, Silvia Baiguera, Costantino Del Gaudio

**Affiliations:** ^1^E. Amaldi Foundation, Via del Politecnico, 00133 Rome, Italy; ^2^Research Center for Regenerative Medicine, University of Rome Tor Vergata, Via Montpellier 1, 00133 Rome, Italy

## Abstract

Raman spectroscopy is a powerful optical technique based on the inelastic scattering of incident light to assess the chemical composition of a sample, including biological ones. Medical diagnostic applications of Raman spectroscopy are constantly increasing to provide biochemical and structural information on several specimens, being not affected by water interference, and potentially avoiding the constraint of additional labelling procedures. New strategies have been recently developed to overcome some Raman limitations related, for instance, to the need to deal with an adequate quantity of the sample to perform a reliable analysis. In this regard, the use of metallic nanoparticles, the optimization of fiber optic probes, and other approaches can actually enhance the signal intensity compared to spontaneous Raman scattering. Moreover, to further increase the potential of this investigation technique, aptamers can be considered as a valuable means, being synthetic, short, single, or double-stranded oligonucleotides (RNAs or DNAs) that fold up into unique 3D structures to specifically bind to selected molecules, even at very low concentrations, and thus allowing an early diagnosis of a possible disease. Due to the paramount relevance of the topic, this review focuses on the main Raman spectroscopy techniques combined with aptamer arrays in the label-free mode, providing an overview on different applications to support healthcare management.

## 1. Introduction

The phenomenon of light inelastic scattering was firstly postulated by Smekal in 1923 and experimentally observed by Raman and Krishnan in 1928. In the original experiment, sunlight was focused onto a purified liquid by a system of various lenses, and several optical filters were used to demonstrate that the collected scattered radiation emerged from the sample with a different frequency from that of the incident light. This phenomenon was then referred as Raman spectroscopy [1].

**Figure 1:**
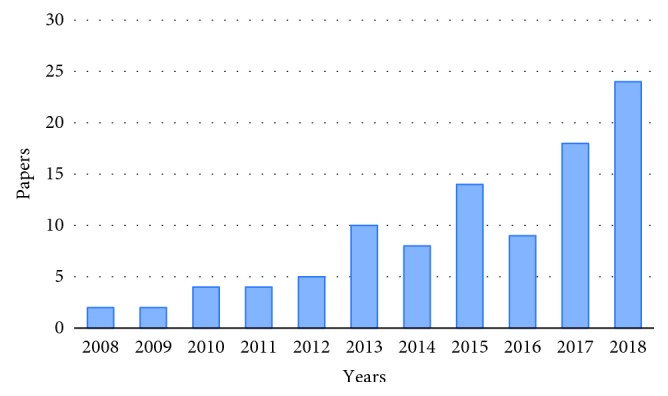
Number of published papers as resulted from the PubMed database (keywords: “aptamer Raman SERS”).

Raman spectroscopy uses a monochromatic radiation, in the range of near IR or UV/Vis, to scan the sample and detect the radiation scattered from its molecules, which is a representative of photon excitation to a virtual energy state, resulting in an energy loss or gain. This energy shift is a characteristic of discrete vibrational modes of polarizable molecules and gives qualitative information about sample chemical composition and bounds. As energy levels at specific frequencies are typical of different functional groups, Raman spectra can also be used to quantify the sample chemical groups: the intensity of a certain peak results, indeed, directly proportional to the concentration of functional groups (usually, a calibration procedure is needed to determine the relationship between peak intensity and concentration). In Raman scattering, the molecules, irradiated by a monochromatic source, experience a polarization of the electron cloud which leads to an unstable short-live state, called “virtual state.” This occurrence is related to an inelastic process, which is inherently weak and occurs with a low probability. As a consequence, in order to perform a reliable analysis, it is necessary to deal with an adequate quantity of the sample, especially when biological ones need to be investigated, and this is not always possible. A potential approach to overcome this limitation can include the use of aptamers, a suitable means that allows to detect a specific target with a high selectivity and even at very low concentration. In the previous years, not many papers have been published on this topic (Figure 1), underlining, on the one hand, the increasing interest in the field and also suggesting, on the other one, that a great effort is still needed in order to fully exploit the potential of this experimental approach. In fact, the expected outcome is not straightforward to be acquired as it might seem, due to the necessary and demanding univocal identification of the spectral features of the aptamer-target complex to be assessed. An intuitive and simple method to support the analysis is then related to the modification of the aptamers by adding a label which is characterized by a well-known Raman spectrum and can thus contribute either to identify or quantify the target. However, also this method can show possible drawbacks as further processing steps are needed and might affect the sample, especially biological ones [2].

These opening considerations suggest that, even if it is possible to deal with a powerful technique, a number of constraints should be carefully evaluated as well, considering the possible influence of multiple factors on the final result. In particular, this review focuses on the studies presented so far, in which the aptamer label-free approach in Raman spectroscopy was presented. The aim is to investigate on the potential of this specific experimental methodology in different fields for diagnostic purposes, being the one that implies a limited manipulation of the sample to be evaluated and that should, therefore, minimally introduce other variables among which discriminate. At the same time, possible limitations and the related strategies that can be planned, if possible, to obtain a feasible detection will be also addressed.

## 2. Raman Techniques

Raman spectroscopy has paved the way for the development of new and ad hoc analytic methods to improve the performed analysis, such as coherent anti-Stokes Raman scattering (CARS), surface-enhanced Raman scattering (SERS), and surface-enhanced resonance Raman scattering (SERRS), and spatially offset Raman spectroscopy (SORS) (Table 1).

CARS is a widely used nonlinear technique based on the excitation of more laser sources, typically three different laser sources. The first beam provides the virtual state of Raman scattering; the second one is characterized by a frequency that should be equal to that which would be scattered in spontaneous Stokes Raman scattering, and the third laser excites the molecule to a second virtual state. Inducing coherent molecular vibrations, this technique increases the speed and the spatial resolution of Raman spectroscopy. CARS allows the imaging of biological tissues, and it has been mostly used for cell observation, by exciting the CH stretching vibrational mode in lipids and proteins in the region between 2600 and 3000 cm^−1^ [3].

SERS is an interesting alternative of Raman spectroscopy that integrates traditional techniques and improves simplicity in terms of samples treatment, sensitivity, and readiness. Thanks to different analytical models that can be implemented to evaluate the information content in spectra and to the absence of the influence of aqueous environment of biological systems, this technique allows to acquire fingerprint signals of vibrational modes that correspond to the univocal identification of the sample. SERS, based on the use of metallic nanostructures (such as Au, Ag, or Cu nanoparticles), enhances the Raman scattering up to about 10^6^, and this occurrence was initially observed by Fleischmann et al. [9]. Indeed, when molecules of interest are absorbed or located near metallic nanostructures, Raman scattering is amplified due to the resonant interactions of the source with the surface plasmons of metallic structures themselves [10]. SERS can overcome the Raman limitation related to weak signals and the necessity of large amount of sample, and it represents a suitable approach to investigate specific specimens due to the increasing of the signal intensity [5].

SERRS combines the advantages of surface enhancement with the use of a resonant chromophore. The intrinsic effect of this technique is elicited when the analyte has a chromophore close in energy to the frequency of the source used to excite the plasmon; enhancement from both plasmon resonance (SERS) and molecular resonance from the analyte effectively contributes to give very intense scattering. Mahajan et al. [7] demonstrated for the first time the SERRS effect on electrodeposited gold films templated with colloidal spheres, also showing the reproducibility of the response.

SORS technique, allowing spectral measurements from volume samples as deep as 10–20 mm, overtakes the limit of Raman spectroscopy that investigates only a few hundred microns as maximum penetration depth, as a spatially offset measurement collects multiple measurements of the scattered light away from the point of laser illumination. This technique is extremely suitable in clinical analysis of tissue slides, including bone disease [8, 10].

The expected output of these techniques can be further improved when opportunely coupled with specific means to optimize the experimental investigation of a sample, especially if intended for diagnostic purposes. Reasonably, a more than confident spectral analysis related to the identification and quantification of the analyte of interest is therefore needed. In this regard, aptamers have been considered as a valuable new strategy to overcome Raman limitations.

## 3. Aptamers

The term “aptamer,” derived from the Latin word “aptus” (to fit) and the Greek word “meros” (part), was first introduced in 1990 to describe RNA molecules that bind to a small organic dye [11]. They were discovered almost simultaneously by the research groups of Ellington and Szostak [11] and Tuerk and Gold [12].

An aptamer is a synthetic, single, or double-stranded DNA or RNA oligonucleotide (generally 15–60 bases in length) that, folding up into a unique 3D structure, is able to specifically bind to the target of interest (mostly protein) through conformational recognition. This peculiar behavior contributed to the “popularity” of these kind of oligonucleotides as capture agents, being adaptable to various targets, convenient in screening, reproducible for synthesis, and versatile in labelling, immobilizing, and signaling [13, 14]. In addition, recent technological advancements have led to a faster and cheaper production of new aptamers, particularly through the SELEX (systemic evolution of ligands by exponential enrichment) protocol, aimed to find an aptamer that binds to the active site of the target molecule [15]. A random DNA or RNA oligonucleotide library pool (containing between 10^13^ ÷ 10^15^ members) is firstly incubated with the target of interest. In the case of RNA SELEX, the library of single-stranded RNA molecules is prepared by *in vitro* transcription of double-stranded DNA templates, while for DNA SELEX, the library of single- or double-stranded DNA molecules is often prepared by the strand separation of double-stranded PCR products. The SELEX process is based on the ability of specific small oligonucleotides, folded in a unique 3D structure, to interact with a target protein with high specificity and affinity, by means of Van der Waals surface contact or hydrogen bonding. Generally, many rounds of SELEX are required to isolate aptamers with the highest selective affinity to the target, and a negative selection with a control target is often required. After a washing step, to separate and remove nonbinding oligonucleotides, the binding sequences are subsequently amplified with PCR (for DNA aptamers) or reverse transcription-PCR (for RNA aptamers). The process is repeated until the pool is enriched for the sequences that specifically recognize the target and, finally, the enriched pool is cloned and sequenced to obtain the individual sequences of interest. A complete SELEX process comprises between 8 and 16 cycles.

Aptamers exhibit high affinity for their targets, and the dissociation constant *K*_d_ of the resulting complexes typically range from low micromolar to high picomolar values [16]. They are also able to discriminate between closely related isoforms or different conformational states of the same molecule [17] and retain their binding and inhibitory activity even after immobilization on carrier materials [18]. In addition, aptamers are different from antibodies not only because they are derived from an *in vitro* process and are characterized by a broader range of functions in analytical applications, clinical diagnoses, and therapeutics, but also, conversely from antibodies, they are also prone to bind to functional domains of the target protein, in particular substrate binding or allosteric sites, thereby modulating the biological function of the molecule [19]. Moreover, still compared to antibodies, aptamers are nonimmunogenic, and the chemical production process is not exposed to viral or bacterial contamination. However, as therapeutic agents, the main limitation of aptamers is related to pharmacokinetic and other systemic properties that are variable and hard to predict [20].

Several aptamers have been generated against a wide variety of targets ranging from small molecules [21], peptides [22], amino acids [23], and proteins, including cell membrane proteins [24]. However, despite the large number of aptamers designed for many different molecules, only a few have been used. Many publications on aptamer-based assays refer to only a limited selection of aptamers: thrombin-binding aptamers represent the most used ones for biosensing, followed by ATP (adenosine-5′-triphosphate**)** aptamers, PDGF (platelet-derived growth factor) aptamers, IgE (immunoglobulin E) aptamers, cocaine aptamers, lysozyme aptamers, theophylline aptamers, and VEGF (vascular endothelial growth factor) aptamers [25]. This occurrence might be related to the fact that those aptamers are now well defined and therefore commercially available and cheaper. The real challenge when dealing with aptamers is mostly dependent on their first synthesis and optimization, which still represents a complicated and expensive process.

## 4. Label-Free Approach

Once a tailored experimental setup is accurately designed, including the selection of a Raman spectroscopy technique and the binding aptamer for the specific analyte, another possible concern can be raised. The detection of the resulting spectrum should be unique and unambiguous for the sample under investigation in order to provide a clear interpretation of the data. This is a crucial step that needs to relate the collected spectrum only to the aptamer-analyte complex, which is a particularly tricky task to perform to avoid any labelling procedure that could simplify the study, but introduces a number of limitations as well. In a typical biosensing process, molecular interactions are transduced as mechanical, electrical, or optical signals and are potentially detectable without any label probes. Moreover, it should be underlined that it is not always possible to label a sample, and it is often necessary to deal with a sample as it is, which is of primary importance when dealing with biological markers.

In the indirect detection approach, the target molecule is labelled with a reporter molecule or a sandwich structure is made, and the concentration of the target molecule is determined through the signal obtained from the label molecule. Fluorescent labels are often considered for Raman spectroscopy, and even if they support the implementation of a very sensitive analysis, their use does not provide information about the chemical composition and the molecular identification of the sample [26].

Label-free detection presents a number of advantages, allowing, for instance, tracking molecular events in a real-time manner and thus permitting to acquire more direct information of the sample. Moreover, this methodology removes additional measurement uncertainties since the acquired signal derives exclusively from the molecular structure of the target of interest. However, drawbacks of a label-free detection in Raman spectroscopy can be highlighted as well, such as the possible not straightforward information derived from the spectra of the specimen under investigation (if totally unknown, but this could be a rare occurrence) and the correct interpretation of collected data.

## 5. Applications and Diagnostic Tools

### 5.1. Bacteria and Spores

In recent years, large efforts have been provided to identify different bacterial species with pathogenic implications. The detection, evaluation, and quantification of pathogenic bacteria are of fundamental importance due to the correlation between several serious and fatal medical conditions and infections. The routinely common detection mode includes cell culture-based methods and immunoassays that are time-consuming and very expensive [27, 28]. Moreover, for some slow-growing organisms, several weeks are needed to obtain the definitive microbiological diagnosis, while some organism cannot be cultured at all and false negative results may occur due to viable but nonculturable bacteria [29].

An ideal bacteria-detection method should be highly sensitive and specific, easy to operate, and not based on time-consuming culture procedures. The use of aptamers, capable to assemble into well-defined bacteria-aptamer complexes, has been recently proposed for SERS bacteria detection and resulted to be a quick, direct, and efficient label-free Raman strategy, offering significant advantages over existing approaches. Using specific aptamers coated with silver nanoparticles, Gao et al. [30] demonstrated that the bacteria SERS signal was dramatically enhanced by specifically recognized aptamer and that the bacteria could be identified directly through the SERS spectrum. Inspired by *in situ* coating Ag nanoparticles with bacteria, a direct label-free detection of *Staphylococcus aureus, Salmonella tiphirium,* and *Shigella flexneri* was developed. In particular in the work of Mello et al. [31] the main microorganisms responsible for gastroenteritis were studied. With traditional methods, the time required for such identifications can be computed in several days, and it could have serious consequences in terms of clinical outcome, especially when the patients are children, elderly, or adults with low resistance [31, 32].

Based on aptamer capture, He et al. [33] developed an innovative label-free platform capable to detect *Bacillus anthracis* spores in orange juice and to discriminate between spores of *Bacillus anthracis* and *Bacillus mycoides* within a short time period (40 min). Among 92 DNA aptamer sequences, BAS-6R and 6R aptamers resulted to be useful for rapid detection of anthrax in complex matrices (e.g., food) and allowed to identify three peaks (fingerprint peaks) of the spores, the results of which correlated with the contaminant concentration [34]. Negri et al. [35] described a SERS-based method for the detection of influenza viral nucleoproteins. A polyvalent influenza aptamer was modified at 5′ with C6-disulfide group and then immobilized on Ag nanorods; the collected spectra showed a high specificity to characterize influenza viral nucleoproteins.

Other authors adopted SERS technique and aptamers for pathogen bacteria detection, including different modified molecules as Raman reporter; this approach favors a simpler spectral investigation. Zhang et al. [36] developed a biosensor for the simultaneous detection of *Salmonella typhimurium* and *Staphylococcus aureus.* Using the specific aptamers coupled with Fe_3_O_4_ magnetic gold nanoparticles, as capture probe, and gold nanoparticles modified with Raman molecules (mercaptobenzoic acid and 5,5′-dithiobis(2-nitrobenzoic acid)) and aptamers, as signal probe, characteristic peaks of the two molecules were detected and correlated to bacteria concentration. The Authors demonstrated that the method is simple and rapid, results in high sensitivity and specificity, and suitable to investigate real samples.

### 5.2. Toxins and Organic Compounds

Contaminated food with harmful pathogen, toxins, or organic pollutants, like pesticides, can be likely correlated to more than 200 diseases annually [37]. Traditional methods to detect contaminants in food are often inadequate and expensive; in this scenario, Raman spectroscopy associated to modern biotechnology can be usefully considered.

#### 5.2.1. Ochratoxin A (OTA)

OTA is a mycotoxin produced by various microorganisms commonly found in food like cereals, coffee, beer, wine, and grape juice. OTA is a stable compound, not affected by common food preparation procedures, which can be neutralized by a heating process above 250°C for several minutes [38]. The maximum OTA concentrations in foods must not exceed 5 ppb in cereals, 2 ppb in wine, and 0.5 ppb in infant food (European Food Safety Authority, 2006).

Currently, for food quality controls, ELISA (enzyme-linked immunosorbent assay) is used with a limit of detection of 0.5 ppb; however, the assay requires the use of monoclonal antibodies, long detection time, and it is very expensive [39]. Recently, Gillibert et al. [40] proposed a fast and sensitive method to detect OTA based on SERS and aptamers in a label-free approach, using nanostructurated substrates. OTA was detected at a picomolar range, much lower than the minimum legal concentration in food products. SERS signal intensities of specific spectral bands decreased with OTA concentration probably due to the interaction with the related aptamer. In addition, some spectral features, observable if OTA only is analyzed, were not visible when the OTA-aptamer complex formed, and this could be directly ascribed to the different configurations that could induce drastic changes in the spectral signature of the target.

#### 5.2.2. 3,3′-4,4′-Tetrachorobiphenyl (PCB 77)

Polychlorinated biphenyls (PCBs) are a class of persistent organic pollutants that can cause significant toxicity due to their bioaccumulation [41]. Several methods have been developed to monitor and quantify PCBs, like electrochemistry [42] or gas chromatography/mass spectrometry [43]; however, the analysis in food samples presents a number of difficulties because of the specificity of the protocol to be applied to prepare the sample itself, consisting in multistep purification stages (the major concern implies the analyte enrichment and the removal of interfering substances). Clearly, the correct implementation of all steps for sample preparation is crucial for the reliability of the final result [44]. Raman analysis in SERS approach may overcome these drawbacks because it potentially does not require any preparation of the samples.

Lu et al. [45] proposed a label-free approach to detect PCB 77 using a specific aptamer and a SERS substrate made by silica modified with Au nanoparticles. The fabrication of the SERS substrate was previously presented by Oldenburg et al. [46]. The authors evaluated the intensity decrease of the ratio of two peaks (660 cm^−1^/736 cm^−1^), which allowed to measure trace amounts of PCB 77 in a selective and quantitative way. The characteristic peaks of PCB 77 and those of the resulting PCB 77-aptamer complex were directly related to the analyte concentration, while the other authors showed an indirectly correlation between analyte concentration and diagnostic band intensities [40].

PCB 77 label-free detection was also performed by Fu et al. [47] by means of a device using an aptamer-based SERS microfluidic sensor. SERS intensity decreased with PCB 77 concentration, and the experimental setup provided several advantages, thanks to the small volume of analytes needed, which is meaningful for biological samples (the lowest detectable concentration of this aptasensor was as low as 1.0 × 10^−8^ M).

#### 5.2.3. Pesticides

Due to the increase application of pesticides, the development of a quick, easy, cheap, effective, and safe detection technique has become a hot topic in recent years [48]. Pesticide residues with similar structure, especially organophosphorus compounds, are difficult to be separated and identified in complex matrices, like beverage or food. Currently used methods, mostly chromatography (i.e., gas chromatography or liquid chromatography) coupled with mass spectrometry, have several disadvantages including extensive sample preparation, technical expertise, and high costs. SERS combined with aptamer technology, once again, is a possible promising strategy to be implemented for the detection and discrimination of these compounds. Moreover, this approach results to be more economic with respect to those currently and largely used, i.e., high-performance liquid chromatography [49].

Pang et al. [50] evaluated the potential of the aptamer-based SERS technique for pesticide detection in a complex liquid food using a single aptamer that was previously synthesized to be specific not for one, but for four commercially available pesticides (isocarbophos, omethoate, profeonofos, and phorate). Resulting spectra, however, showed a very poor difference, being necessary to consider the second-derivative Raman spectra to discriminate the signals of interest in a sample including the four targets. The authors showed that each pesticide is characterized by distinct peaks at different Raman shifts, demonstrating that a pesticide discrimination can be carried out. Furthermore, they demonstrated that the Raman peak changes attributed to pesticides could be statistically quantified and differentiated, suggesting that this method could identify four different targets within one sample. However, the total pesticide concentration for each sample was 0.5 mM, and the sensitivity of this SERS method resulted to be slightly lower with respect to currently used techniques, considering the initial high concentrations. Moreover, the limit of detection of each target resulted very different although the reported binding dissociation constants were similar for the selected pesticides. The results demonstrated the greater capacity of aptamer-based SERS in rapid detection and discrimination of multipesticides, even if this technique can be improved by using an aptamer with higher binding dissociation constants.

Nie et al. [51] proposed a novel label-free aptamer SERS sensor for trace malathion (an organophosphate pesticide widely used in agriculture) residue detection. The experimental protocol implemented silver nanoparticles (AgNPs) treated with spermine (Sp) to form a highly stable suspension due to the resulting positive surface charge (AgNPs@Sp), as already reported by Masetti et al. [52]. Metallic nanoparticles combined with the aptamer-malathion complex due to the negative phosphate backbone of the aptamer, which electrically interacted with the positive spermine. In this regard, it can be also underlined that the aptamer was modified in 5′ with a thiol function and, maybe, the bound with AgNPs@Sp cannot be totally ascribed to an electrostatic interaction, but also to the thiol affinity to the Ag surface. The characteristic peak of malathion at 1112 cm^−1^ changes with its concentration, allowing to detect a value of 5.10^−7^ mol·L^−1^ as lower limit. The effective specificity of the considered aptamer for malathion was confirmed, considering possible pesticides interference molecules, such as phosmet, chlorpyrifos-methyl, and fenthion. The authors suggested that the proposed label-free aptamer SERS sensor is convenient, specifically detects trace malathion residues, and can be applied for qualitative and quantitative analysis of other pesticides.

### 5.3. Proteins

The assessment of protein biomarkers for diagnostics is another field in which Raman spectroscopy can be usefully considered as a suitable tool to be integrated with routinely assays to promote an early detection and eventually improve the therapeutic protocols. Nowadays, there are many commercial kits for protein analyses, but their application in diagnostic centers and daily use poses severe limitations. For example, ELISA is one of the most common methods for clinical protein determination, assuring a good detection limit for some protein biomarkers; however, it requires significant technical expertise and suffers in terms of time analysis, equipment costs, and sample amount [53].

Multilabel detection seems suitable to evaluate a wide range of protein concentrations in a range of sensitivity of pg·mL^−1^, and multilabel arrays can be designed to detect high and ultralow abundance proteins in the same sample. However, only few recent techniques have been evaluated with real patient samples, in order to validate the method and to establish clinical sensitivity and selectivity [54]. It should be underlined that there are thousands of proteins at significant concentrations in serum, some higher than ng·mL^−1^ levels [55], and ad hoc strategies need therefore to be developed to measure both high and low concentrations of the biomarker panel in the same sample, without interference from thousands of other serum proteins (many at higher concentrations than the target analytes). Moreover, it should be also highlighted that labelling is not always possible, especially in real patient samples.

Label-free SERS method thus offers a proper alternative, even if it may be affected by possible limitations due to the spectra interpretation and discrimination among different proteins as they often contain the same functional groups in a similar structure.

#### 5.3.1. Human *α*-Thrombin

Thrombin is an allosteric serine protease, which has a crucial role in physiological and pathological coagulation cascade and is involved in various diseases, such as Alzheimer's disease and cancer [56–58]. In normal condition, it is present in the blood only in the inactive form, i.e., prothrombin, which is secreted at a concentration of 1.2 *μ*M. During coagulation, the concentration of thrombin may vary from pM to *μ*M levels [59].

Currently, in the medical practice, thrombin is not directly measured or quantified, and the index used to infer on the clotting blood tendency is the measure of prothrombin time, which provides a temporal parameter for this occurrence. However, several medical cases require an evaluation of blood thrombin levels, as for patients with coagulation factor deficiency (e.g., haemophilia A), cardiovascular diseases, or acquired conditions, like pregnancy which may expose to different thrombosis risks, depending on additional genetic and environmental modulators. The need for global assays, evaluating the overall coagulation function in order to reliably estimate the bleeding/thrombosis risk in each individual patient, is therefore highly desirable. In the 1950s, a thrombin generation assay was developed in order to measure the characteristics of a plasma sample to generate thrombin following *in vitro* activation of the coagulation cascade with tissue factors or other triggers. Differently from classical clotting assays (i.e., prothrombin time), which only probe the starting phase of coagulation, thrombin generation assays also probe the propagation and termination phases [60]. As a consequence, there is a real clinical need to develop a method able to identify and quantify thrombin in a real blood sample.

An assay able to measure low-level thrombin concentration needs to be characterized by a sufficiently low detection limit in an appropriate value range. Several assays have been developed for the detection and the quantification of thrombin, such as clotting-based assays [61], enzymatic activity-based assays [62], immunoassays [63], and aptamer-based methods [64]. In this scenario, and according to the latter approach, the development of label-free aptasensors suitable for SERS assessment could integrate and optimize the obtained results.

The first aptamer binding to human *α*-thrombin was described by Bock et al. [65]. The 15-mer DNA oligonucleotide (TBA15, 5′-GGT TGG TGT GGT TGG-3′) forms a stable intramolecular G-quadruplex structure, which is in an antiparallel orientation with a chair-like conformation [66]. The aptamer interacts with one of the two thrombin anion-binding sites, the fibrinogen-recognition exosite, with a dissociation constant *K*_d_ of ∼100 nM. Another thrombin-binding aptamer is a 29-mer DNA oligonucleotide (TBA29, 5′-AGT CCG TGG TAG GGC AGG TTG GGG TGA CT-3′), which binds to the thrombin heparin-binding exosite with a higher affinity (*K*_d_ = 0.5 nM) [67]. These aptamers bind only to *α*-thrombin and not to *β*- or *γ*-thrombin because the binding sites, i.e., exosite I and exosite II, are partly or fully lost due to proteolytic cleavage of *α*-thrombin.

Pagba et al. [68] focused on the sensitivity of the SERS technique for the direct and label-free detection of thrombin using the TBA15. Silver nanoparticles were immobilized onto a glass substrate and functionalized with a thiolated aptamer, and the surface was protected from the formation of nonspecific binding by a blocking agent like 6-mercaptohexanol. The same research group [69] also reported a SERS characterization study of TBA15, demonstrating the formation of the quadruplex structure of the aptamer (Raman peak at 1480 cm^−1^) which was strictly related not only to the presence of thrombin but also to the high concentration of K^+^ ions. This occurrence allowed to separately identify the characteristic peaks because the SERS spectra were collected in each experimental step to monitor the binding events. The presence of thrombin resulted in additional bands in the spectra, corresponding to the peak at 1140 cm^−1^, the C-N stretching, and at 1540 and 1635 cm^−1^ ascribable to protein amide I and amide II, respectively. Several bands shift and the observed three-fold increase in intensity of the C-H peak, upon addition of thrombin, strongly suggested the binding of the target molecule.

A similar study was reported by Ochsenkühn and Campbell [70], in which gold nanoshells were used instead of silver ones, in a way that some peaks shifted with respect to the signals found with silver despite they investigated the same molecule. Some shifts of the peaks are considered normal when using different metal nanoparticles as enhancer; however, different laser lines should not give differences examining the same materials, but often these differences occur. Indeed, in this work, the Authors identify the peaks ascribable to the complex thrombin-aptamer at 822 cm^−1^, 1140 cm^−1^, and 1558 cm^−1^, assigned to the combined C2′-endo and C3′-endo modes of the 2′-deoxyribose sugars, the C–O–C stretch at 1140 cm^−1^ and guanine ring modes at 1558 cm^−1^. Sensor reproducibility and reusability was also verified: successive washing steps performed with buffer solution disrupted, indeed, the aptamer-protein interaction.

More recently, Scatena et al. [71] investigated the interaction of thrombin with TBA15, developing a different approach. The proposed aptasensor was prepared by functionalizing a gold nanostructured substrate, as a reliable means to implement the SERS technique. Differently from sensors so far presented, this one was assessed in dry conditions, ensuring the unambiguous detection of thrombin at very low concentration. The experimental set-up is crucial to develop, for instance, a simple and attractive analytical platform for technology transfer purposes when it is not possible to afford expensive Raman spectrometer with immersion objective, which is the commonly used methodology to be implemented for liquid sensing. The SERS analysis was carried out by mapping the surface of the aptasensor, considering the representative bands of aptamer-thrombin previously reported [70], and then combining them to acquire unambiguous measure of the target. A schematic summary of these studies is reported in Table 2.

Thrombin is a protein of particular interest, and it can be considered as a relevant model deeply studied, especially referred to aptamer bonding. This topic was specifically investigated in order to give a better insight of Raman spectra, focusing on Raman signals of aptamers only, before or without adding the target molecules. Pagba et al. [72] investigated on the conformational changes in the quadruplex structure of the 15-thrombin-binding aptamer and in others guanine-rich oligonucleotides. A quadruplex structure is formed by guanine-rich residues by intramolecular folding or by intermolecular association via Hoogsten-type guanine-guanine interaction. The quadruplex structure is firstly found in telomeres, a repetitive nucleotide sequences in chromosome, and in several proto-oncogenes structures, such as VEGF, and it is of particular interest as they are potential molecular target of anticancer drugs [73, 74]. It was found that the temperature and the chemical environment have a critical role to maintain the quadruplex structure stability. The conformational changes are confirmed in the Raman spectra by the decrease of the intensity of the peak at 1480 cm^−1^ that is assigned to the C8 = N7‐H2 bond deformation of the guanine tetrad and is a marker of the quadruplex structure [72].

The SERS detection of biological species is not easy if compared with the detection of inorganic ones; it is particularly challenging for biomolecules with multiple chemical bonds and a large variety of possibly conformations [75–79]. Wu et al. [80] studied the SERS signal of TBA15 with a six-base spacer in 3′ (5′ GGT TGG TGT GGT TGG ATT TTT3′), firstly on a substrate made of a silver layer (thickness from 100 to 200 nm), coated on polystyrene nanospheres (390 nm), deposited on a glass coverslip and then on a different substrate in which double-stranded TBA sequences were sandwiched between 1.4 nm gold nanoparticles and a 50 nm gold thin film, and deposited on a glass coverslip as well. A comparison of two samples was performed, one consisting of 5 *µ*M TBA in KCl, and the other one of TBA 10 *µ*M sequence alone. The presence of potassium did not cause any peak shift, while the major difference was the presence of the peak at 1096 cm^−1^ that was ascribable to the phosphate backbone of DNA. The purpose of these two formats was to infer on SERS spectra of the TBA aptamer sequence for two different geometrical configurations of nanostructures eliciting the SERS effect. In this study, the peak at 1480 cm^−1^ was considered as indicative of the quadruplex, but it was reported that the peak of the backbone at 1096 cm^−1^ without K^+^ ions and the one at 1100 cm^−1^ with K^+^ ions were representative of the bending of the backbone to form the quadruplex structure, which in turn causes the vibrational frequency to change. The characteristic peaks of the guanine were identified at 656 cm^−1^ (the primary peak) and four minor peaks from 1300 to 1550 cm^−1^, and it was noted that the spectral features shifted in a range 5–21 cm^−1^ with respect to known values from conventional Raman measurements.

#### 5.3.2. Lysozyme

Lysozyme is one of the major egg allergens, and its identification is fundamental to understand gut disease causes. Food allergy in fact is an immunogenic response that can cause several adverse effects also associated with a relevant reduction of life quality [81], and the assessment in this field is thus important to contribute to prevent avoidable allergic reactions.

Boushell et al. [82] proposed an aptamer-based sensor for the detection of lysozyme in food-handling surface. A specific aptamer was modified with a thiol function and then immobilized onto silver dendritic nanoparticles for SERS detection. Lysozyme Raman spectrum was acquired as a reference to validate the bands found after the interaction with aptamers. Two prominent Raman peaks were detected at 1008 and 765 cm^−1^, which were additionally validated by separated studies [83, 84].

However, only the peak at 1008 cm^−1^ was selected as an indicator for the capture of lysozyme, which might be a critical detection, especially if the analysis is performed in real samples where more interfering proteins may be present, and the spectral region around 800–1100 cm^−1^ is characterized by the Raman scattering of many compounds.

## 6. Conclusions

This review focused on SERS/Raman analyses carried out to specifically detect and capture several targets, implementing a label-free approach combined to aptamers. This experimental approach is characterized by many advantages, even if it may lead to a difficult interpretation of the acquired spectra caused by the possible signal overlapping in complex macromolecules.

Some authors [40] found that the interaction of the target with the aptamer led to decreasing of the band intensities, while others [70, 71] reported that the formation of the complex did not change the band intensities but rather induced a shift in the position of the diagnostic peaks compared to those of neat compounds. This occurrence implies a particular care when a search match is performed by using predefined libraries. The importance of having a reliable library is a priority, especially in the case of biological molecules which can exist in different conformations or folding, a parameter that can affect Raman signals. Peak assignment is not therefore straightforward and uncertainties cannot be removed and the introduction of a label could be a possible solution that, however, should be carefully considered as it implies the adoption of a specific experimental protocol that could affect the target to be analyzed. In this respect, the label-free methodology, even if more complex, seems to be preferable as a direct approach that reduces the processing steps during the preparation of the sensor and limits the risks to alter the matrix under study, which could be a serious issue when dealing with biological samples for diagnostic purposes.

The potential of the experimental approach discussed here should be further exploited as it can provide a reliable method to carry out extensive and accurate measurements of several compounds directly (or indirectly) affecting healthcare. In this regard, the development of ad hoc setup capable to readily perform an effective evaluation, even in an operational field and not only in a fully equipped laboratory, should be clearly promoted. The latter point is reasonably a valuable and shareable topic to be considered because the high sensitivity of the Raman SERS-aptamer systems can actually support the data collection of harmful agents at very low concentrations and, therefore, allow counteraction with tailored procedures to prevent undesired effects. This perspective should be a goal to be achieved in order to design simpler and fully automated detection instrumentations, to consistently apply a powerful technique, and to increase the level of life quality, as a consequence.

In addition, with the aim to improve a label-free approach and thus reduce the manipulation of a sample, as already underlined, ad hoc protocols should be developed. A possible related research topic could be focused on the optimization of aptamer sequences. Aptamers are often characterized by redundant sequences not involved in the capture of the target, and a suitable design can allow to deal with lighter probes having less folding-unfolding constraints and interference issues during the Raman analysis.

The sensitivity of the technique and the definition of tailored means can actually enhance the healthcare management in order to introduce additional assays that can be integrated with those routinely carried out to provide a detailed and comprehensive diagnostic result.

## Figures and Tables

**Table 1:** Comparison of different Raman derivative techniques.

Technique	Characteristics	Advantages	Fields of application	References
CARS	Use of more laser sources	Increased speed and spatial resolution	Tissues and cells imaging Real-time imaging	[3]
SERS	Use of metallic nanoparticles such as Au, Ag, and Cu	Enhancement of scattering and of signal intensity	Biomolecules Samples available in very small quantities	[4–6]
SERRS	Use of silver colloid solution as aggregating agent	Intense scattering from samples that show inherently IR fluorescence	Forensic examinations	[7]
SORS	Collecting a set of Raman spectra regions away from the point of laser illumination	It allows deeper spectral evaluations within the sample	Clinical diagnostic of tissue slides	[8]

**Table 2:** Schematic summary of studies focused on label-free Raman signal of *α*-thrombin captured by 15-TBA.

Diagnostic peaks (cm⁻ˡ)	Enhancer	Method of analysis	Concentration	References
1140: C-N stretching of THR 1540: Amide II 1635: Amide I	AgNPs	Wet	10 *µ*M	[68]
822: 2′deoxyribose 1140: C-O-C stretch 1150: guanine ring modes	AuNPs	Wet	10 nM	[70]
According to Ochsenkuhn and Campbell [70]	Au nanostructured substrate	Dry	0.1 nM	[71]
